# Visible Light-Induced Specific Protein Reaction Delineates Early Stages of Cell Adhesion

**DOI:** 10.1021/jacs.3c07827

**Published:** 2023-10-31

**Authors:** Rolle Rahikainen, Susan K. Vester, Paula Turkki, Chasity P. Janosko, Alexander Deiters, Vesa P. Hytönen, Mark Howarth

**Affiliations:** † Faculty of Medicine and Health Technology, Tampere University, Arvo Ylpön katu 34, 33520 Tampere, Finland; ‡ Fimlab Laboratories, Biokatu 4, 33520 Tampere, Finland; § Department of Biochemistry, University of Oxford, South Parks Road, Oxford OX1 3QU, U.K.; ∥ Department of Chemistry, University of Pittsburgh, Pittsburgh, Pennsylvania 15260, United States; ⊥ Department of Pharmacology, University of Cambridge, Tennis Court Road, Cambridge CB2 1PD, U.K.

## Abstract

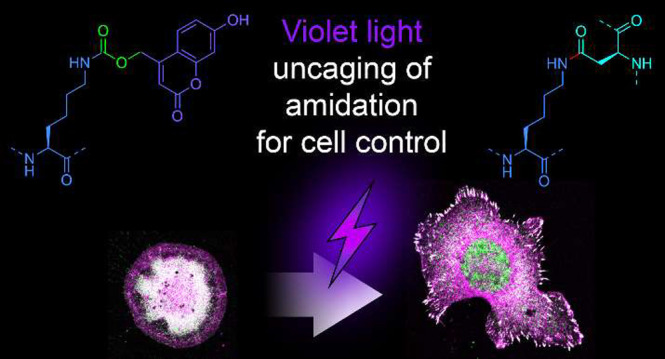

Light is well-established for control of bond breakage but not for control of specific bond formation in complex environments. We previously engineered the diffusion-limited reactivity of the SpyTag003 peptide with its protein partner SpyCatcher003 through spontaneous isopeptide bond formation. This system enables precise and irreversible assembly of biological building blocks with applications from biomaterials to vaccines. Here we establish a system for the rapid control of this amide bond formation with visible light. We have generated a caged SpyCatcher003, which allows light triggering of covalent bond formation to SpyTag003 in mammalian cells. Photocaging is achieved through site-specific incorporation of an unnatural coumarin-lysine at the reactive site of SpyCatcher003. We showed a uniform specific reaction in cell lysate upon light activation. We then used the spatiotemporal precision of a 405 nm confocal laser for uncaging in seconds, probing the earliest events in mechanotransduction by talin, the key force sensor between the cytoskeleton and the extracellular matrix. Reconstituting talin induced rapid biphasic extension of lamellipodia, revealing the kinetics of talin-regulated cell spreading and polarization. Thereafter we determined the hierarchy of the recruitment of key components for cell adhesion. Precise control over site-specific protein reaction with visible light creates diverse opportunities for cell biology and nanoassembly.

Living systems display exquisite precision in their organization and rapid adaptation. Chemical biology aims to exert control over cell or organism behavior, but most methods act over hours to days (genetic modification) or lack spatial control (pharmacological manipulation).^1,2^ However, light allows rapid and precise subcellular responses, e.g., optogenetics to modulate membrane gradients for electrical signaling.^3^ In the area of protein interactions, interactions can be switched by visible light using phytochrome or light-oxygen voltage (LOV) domains.^1^ We have endeavored to develop protein–protein interactions that extend beyond typical stability through genetically encoded irreversible ligation.^4^ SpyTag003 is a peptide that we engineered for rapid isopeptide bond formation with its protein partner SpyCatcher003 (Figure 1A).^5^ Reaction proceeds close to the diffusion limit, occurs under diverse conditions, and is efficient in numerous cellular systems.^5,6^ Tag/Catcher bioconjugation has been employed in biomaterials, vaccine assembly, and antibody functionalization.^4,7−9^ SpyTag003/SpyCatcher003 has also been useful inside cells, including recruitment of epigenetic modifiers or enzyme channeling.^4,10,11^ Previously, an engineered LOV domain allowed photocontrol of SpyTag/SpyCatcher, although there was gradual isopeptide bond formation even in the dark state.^12^ To enable highly switchable covalent reaction, here we employ site-specific incorporation of an unnatural amino acid.^13^ Photoreactive amino acids like benzoylphenylalanine trap complexes after UV activation,^14^ which is powerful to identify unknown complexes but not ideal for targeted bridging.^14^ Individual amino acids can also be photocaged,^13,15,16^ and K31 is the key reactive residue on SpyCatcher003 (Figure 1A).^5^ We focused our efforts on the unnatural amino acid 7-hydroxycoumarin lysine (HCK) (Figure 1B) because uncaging in the visible spectrum (Figure 1C) would reduce phototoxicity that is particularly serious in the UV range.^15,17,18^ Here we establish caging of SpyCatcher003 using unnatural coumarin-lysine amino acid and its uncaging with 405 nm light for spatiotemporal control in living cells to reveal early steps in mammalian cell adhesion.

**Figure 1:**
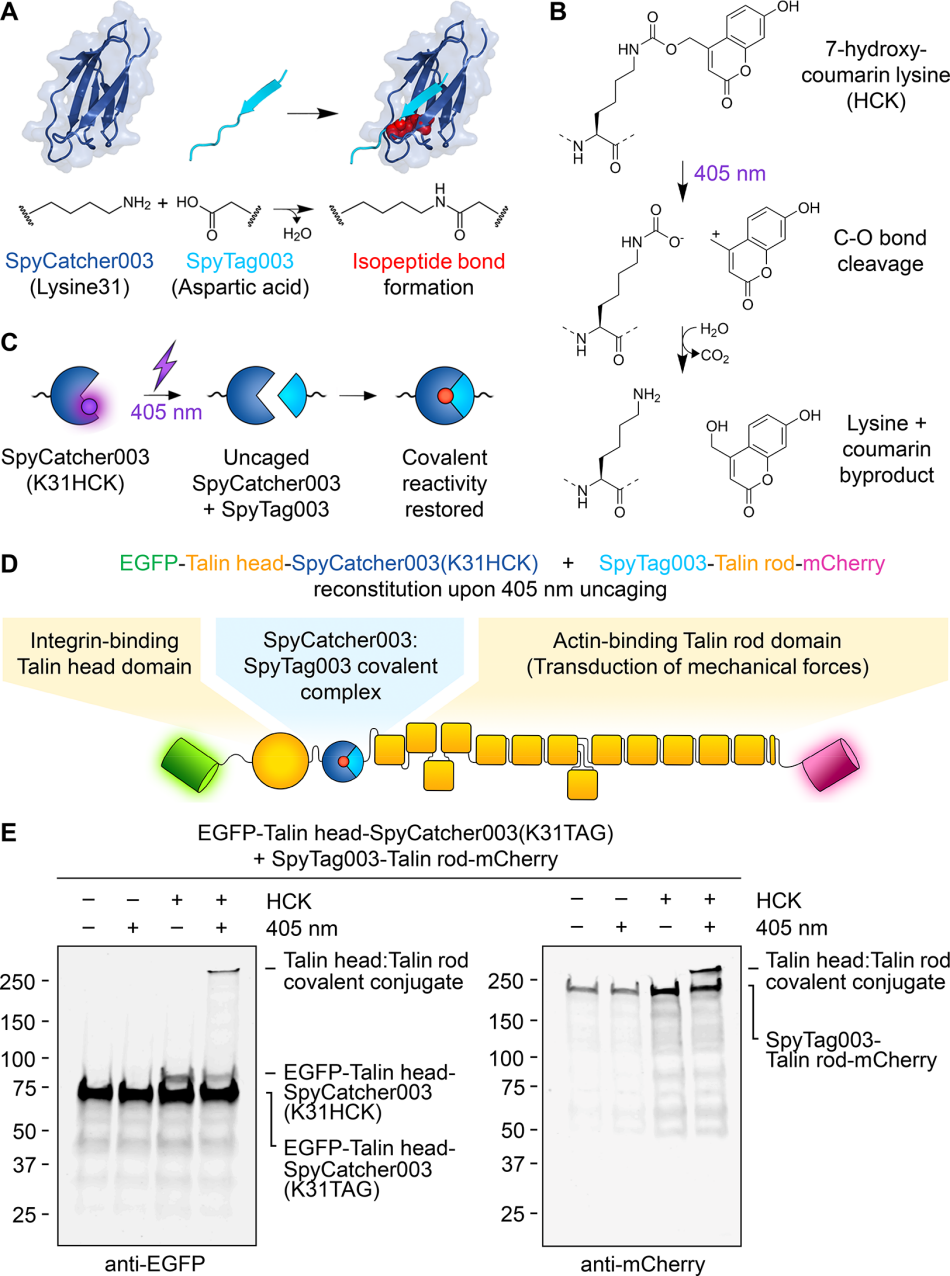
**SpyCatcher003 photocaging with 7-hydroxycoumarin lysine**. (A) Schematic of the SpyTag003/SpyCatcher003 reaction. Lysine on SpyCatcher003 (dark blue) and aspartic acid on SpyTag003 (cyan) form a spontaneous isopeptide bond (reacted side chains shown as red spheres), based on PDB entry 4MLI. (B) Schematic of the light-induced cleavage. Dotted lines indicate the rest of the polypeptide. (C) Schematic for SpyCatcher003(K31HCK) uncaging. (D) Split talin reconstitution using SpyCatcher003(K31HCK). (E) Covalent talin reconstitution upon SpyCatcher003(K31HCK) photoactivation. Talin knockout cells transfected with EGFP-Talin head-SpyCatcher003(K31HCK) and SpyTag003-Talin rod-mCherry were analyzed ± HCK and ± 405 nm light, before Western blotting with anti-EGFP (left) or anti-mCherry (right).

To establish our uncaging approach, we cotransfected the human cell-line HEK293T with HCK tRNAs and HCK tRNA synthetase (HCK RS)^19,20^ along with our protein of interest to show that expression depended on the unnatural amino acid. Our initial construct contained the N-terminal region of transferrin receptor (TfR), SpyCatcher003 with an amber stop codon at K31 (K31TAG), and superfolder green fluorescent protein (sfGFP). Based on Western blotting, we optimized the dose of HCK and the ratio of the SpyCatcher003 construct to HCK RS (Figure S1A).

We then applied a photocontrolled reaction to gain insight into cell adhesion, focusing on talin protein. Talin bridges the cytoplasmic domain of β-integrin to the actin cytoskeleton and functions as a molecular clutch required for actin-dependent cell spreading.^21−23^ Talin changes conformation in response to force, regulating association and release of multiple proteins involved in the cell’s response to mechanical cues.^24^ Talin recruitment has been previously controlled by an elegant strategy using rapamycin as a cell-permeable small molecule to reconstitute FRB- and FKBP-split talin fragments.^25^ However, this approach lacks subcellular spatial resolution and was only tested to withstand force of 4 pN,^25^ which may not resist sustained cytoskeletal tension acting on talin at 10–40 pN.^26,27^ Split talin reconstitution using LOV domains would allow spatial control but depends on continuous 488 nm illumination and has limited interaction stability.^28^ Because of the complex structure and natural turnover of adhesion structures, estimating the impact of such non-covalently reconstituted talin on adhesion function is challenging. Rapid light-mediated induction of covalent talin reconstitution would allow precise control over early phases of adhesion formation to decipher molecular details of talin-dependent processes.

To establish optical control of talin reconstitution, we incorporated SpyCatcher003(K31TAG) in a split talin construct (Figure 1D). We studied HCK- and light-dependent covalent talin reconstitution in fibroblast cells by Western blot against EGFP or mCherry. HCK-caged SpyCatcher003 did not react with SpyTag003 until cells were treated with 405 nm light, consistent with the effective caging of SpyCatcher003 (Figure 1E). We confirmed this tight control of SpyCatcher reactivity also in a different setup, using recombinant SpyTag003-maltose-binding protein (MBP) to probe for SpyCatcher003(K31HCK) reactivity in cell lysates (Figure S1B,C). Western blot with antiserum to SpyCatcher003 (Figure S1B) or anti-EGFP (Figure S1C) demonstrated light-dependent SpyCatcher003(K31HCK) activation and isopeptide bond formation. Without HCK, no SpyCatcher003 expression was detected, indicating that the stop codon led to chain termination (Figure S1B). To understand the practicality for selective uncaging, we assessed uncaging by ambient light. Room lighting or U.K. sunlight for 120 min did not lead to substantial uncaging in cell lysate in microcentrifuge tubes (Figure S2). Depending on the used wavelength and required light dose, optogenetic control of cells even with visible light can lead to phototoxicity.^29^ We confirmed the biocompatibility of 405 nm light on fibroblast cells using a resazurin-based metabolic activity assay. We observed full viability of cells in Trolox-supplemented media even after 3 min of continuous 405 nm exposure (Figure S3).

Having confirmed robust 405 nm photouncaging, we investigated the effects of talin reconstitution in fibroblasts with knockout of both endogenous talin genes.^21^ We transfected with Talin head-SpyTag003 and SpyCatcher003(K31HCK)-Talin rod-mScarletI, along with HCK tRNAs and HCK RS with LifeAct-mNeonGreen to visualize actin. Cells were cultured with HCK and imaged by confocal microscopy with lasers at 405 nm (photoactivation), 488 nm (mNeonGreen, a bright-green fluorescent protein), and 561 nm (mScarletI, a bright-red fluorescent protein). Unactivated cells could not spread or polarize, consistent with the lack of functional talin (Figure 2A).^21,23^ Local photoactivation at 405 nm for 5 s led to lamellipodia extension within seconds (Figure 2B,C and Movie S1), indicating the rapid reconstitution of talin in cells. We did not observe spreading of unactivated cells imaged at 488 nm (Figure 2C and Movie S1), so typical microscopy conditions did not cause unintended photoactivation. Similarly, we did not observe spreading upon 405 nm exposure of cells transfected as described above but with the equivalent DMSO concentration in place of HCK (Figure 2C).

**Figure 2:**
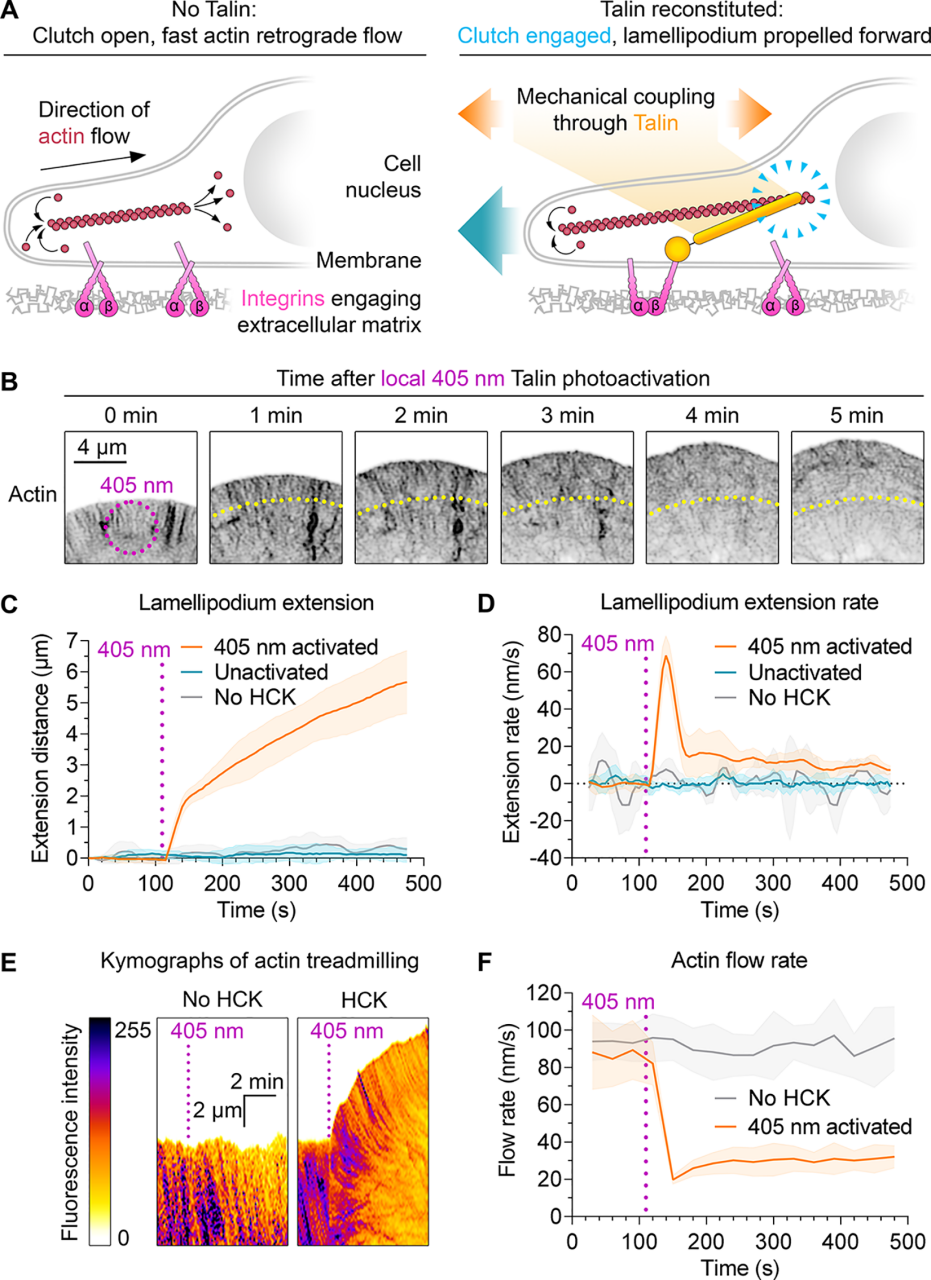
**Photocontrol of SpyTag003/SpyCatcher003 reactivity in living cells**. (A) Schematic of talin’s role as an adhesion clutch. (B) Photoactivation of cell spreading. Talin knockout cells transfected with caged split talin were activated by 405 nm light for 5 s (magenta ring) and imaged at the indicated time points. Inverted LifeAct-mNeonGreen signal for actin is shown. Yellow indicates the original lamellipodium edge. (C, D) Quantification of the lamellipodium extension distance and extension rate. Cells were activated as in (B) and imaged for LifeAct-mNeonGreen. *n* = 5–11 cells. (E) Actin dynamics after photoactivation. Cells were activated as in (B), and kymographs were created for lamellipodium LifeAct-mNeonGreen. (F) Quantification of actin treadmilling. Magenta indicates the point of 405 nm activation. Line represents mean, with shading ±1 SD, *n* = 6–8 cells.

Upon talin reconstitution, we observed biphasic extension of lamellipodia, with a fast initial phase (∼70 nm/s) followed by a slower phase (10–20 nm/s) (Figure 2C,D). Actin polymerization at the cell periphery is the main driving force propelling the lamellipodium forward,^30^ so we investigated actin treadmilling by tracking LifeAct-mNeonGreen (Figure 2E). Unactivated cells had a fast initial actin rearward flow at ∼90 nm/s (Figure 2F). Talin reconstitution led to a sharp drop to ∼20 nm/s, followed by a gradual recovery to ∼30 nm/s (Figure 2F). The sharp drop in actin retrograde flow coincides with the phase of fast lamellipodium extension, suggesting that the integrin–talin–actin clutch is rapidly engaged upon talin photoactivation.

Force sensing by talin generates localized activation of adhesion signaling, regulating cell polarization.^23^ Given the covalent SpyTag003:SpyCatcher003 interaction, this photoactivation strategy should allow extended cell polarization experiments covering tens of minutes. Talin knockout fibroblasts transfected with Talin head-SpyTag003, SpyCatcher003(K31HCK)-Talin rod-mScarletI, and HCK RS plasmids were cultured with HCK and photoactivated with wide-field 405 nm light for 1 min. Cells were fixed at selected time points and analyzed for cell area and morphology. Activated cells showed fast initial spreading and reached close to a maximal area ∼10 min after photoactivation (Figure 3A,B). In contrast, cell polarization was triggered only when the maximal cell area was reached and continued to develop until the end of the experiment (Figure 3C). As expected, cells without HCK did not react to the photoactivation stimulus (Figure 3B,C).

**Figure 3:**
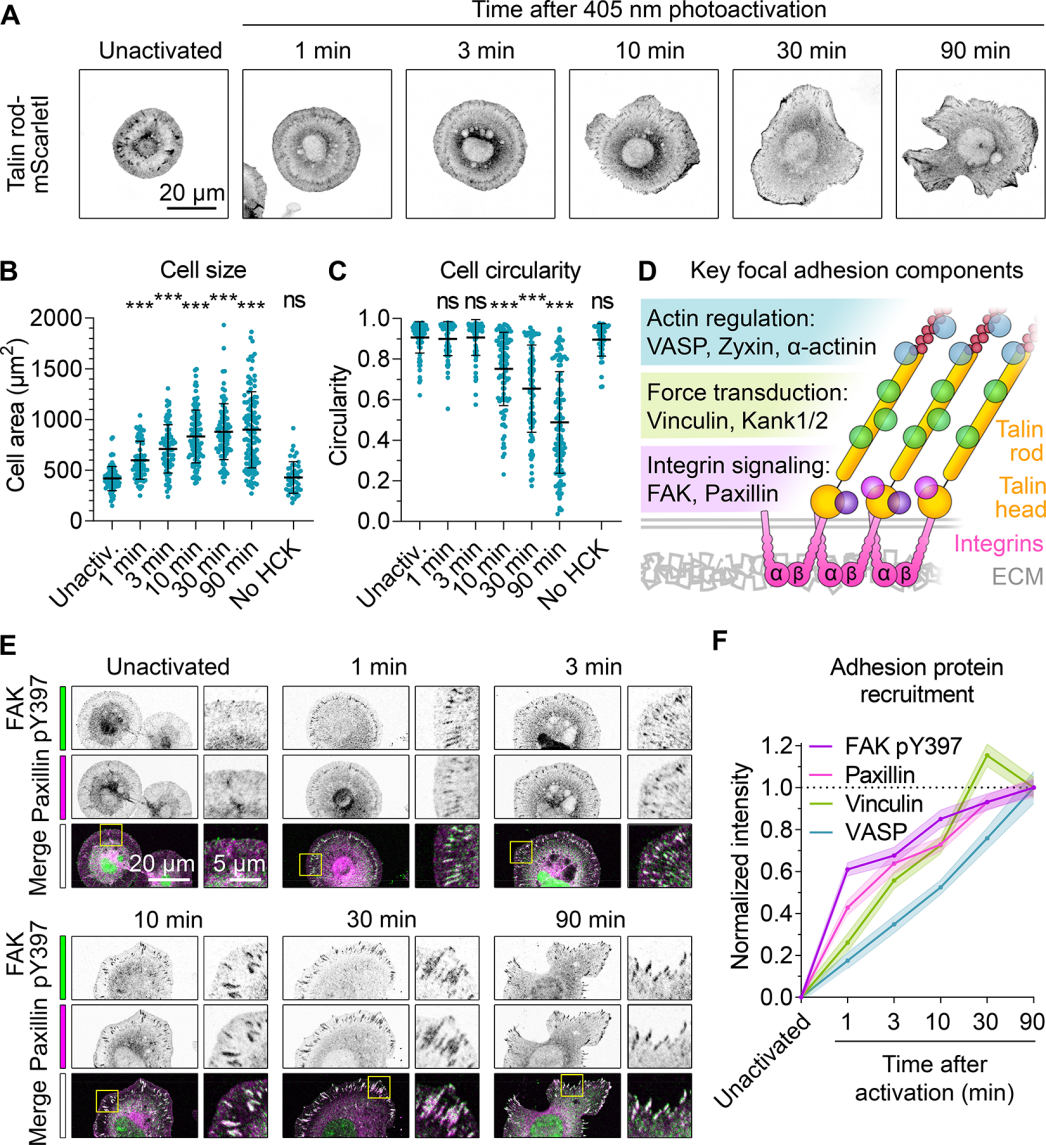
**Photoactivation of talin allows precise control over adhesion complex formation, cell spreading, and polarization**. (A) Cell spreading and polarization after photoactivation. Talin knockout cells expressing Talin head-SpyTag003, SpyCatcher003(K31HCK)-Talin rod-mScarletI, HCK tRNAs, and HCK RS were activated by 405 nm wide-field illumination for 1 min and fixed at the indicated time, before fluorescence microscopy for mScarletI. (B, C) Quantification of cell size and circularity following talin photoactivation as in (A). Each blue circle is one cell, with black lines showing mean ± 1 SD. Compared with unactivated cells using one-way ANOVA and Dunnett’s test: *** *p* < 0.0001, ns *p* > 0.05, *n* = 45–110 cells from two independent experiments. (D) Schematic of key adhesion components. Interactions with talin’s rod domain generate three functional layers (colored bars). (E) Recruitment of adhesion components after talin photoactivation. Talin knockout cells were photoactivated as in (A) and stained with antibodies for fluorescence microscopy (zoom of the yellow square in the right image). Overlap of FAK pY397 (green) and paxillin (magenta) in the merge shows as white. (F) Quantification of recruitment to adhesions following talin photoactivation as in (E). Line represents the mean, with shading ±1 SEM, *n* = 60–85 adhesions in 12–17 cells from two independent experiments.

We next explored the feasibility of fine-tuning SpyTag003/SpyCatcher003 light regulation via single or double amino acid mutations in SpyTag003 (Figure S4A).^5^ We observed reduced spreading of unactivated cells expressing SpyTag003 mutants compared to SpyTag003 itself (Figure S4B,C), suggesting that SpyCatcher003(K31HCK) may form a transient non-covalent complex with SpyTag003 before uncaging. While the SpyTag003(V114T, V116T) complex was unable to mediate stable talin reconstitution and adhesion formation in the absence of light, cell spreading and polarization were equivalent to SpyTag003 after 405 nm activation (Figure S4B,C). SpyTag003(M115G) did allow increased cell spreading after light activation but showed little cell polarization (Figure S4B-D). Therefore, these peptide variants provide alternative properties for light-regulated peptide–protein interaction.

Stretching of talin rod regulates recruitment and release of many adhesion components, generating a structure with distinct functional layers (Figure 3D).^31^ However, the heterogeneous and dynamic structure of adhesion complexes makes it challenging to define the temporal hierarchy of adhesion protein recruitment.^32^ Having validated our method for triggering synchronized adhesion, we investigated the rates of recruitment for key adhesion components. Focal adhesion kinase (FAK) is a central adhesion complex tyrosine kinase that is activated by phosphorylation at tyrosine 397 (pY397).^33^ Paxillin is an adaptor protein interacting with both structural and signaling components.^33^ Vinculin is recruited to mechanically activated sites in the talin rod domain and binds F-actin to reinforce mechanically the adhesion complex.^23,34^ Vasodilator-stimulated phosphoprotein (VASP) is an actin regulator, promoting actin filament elongation through multiple mechanisms.^30^ We observed fast initial FAK pY397 recruitment to adhesions, reaching half-maximal intensity <1 min after photoactivation (Figures 3E,F and S5B). Paxillin and vinculin reached half-maximal intensity at 3 min, with paxillin being slightly faster (Figures 3E,F and S5A,C,D). In contrast, recruitment of VASP reached half-maximal intensity only after 10 min (Figures 3F and S5A,E).

Robust optical control of protein complexation relies on a sufficient bond life of the activated complex, ideally exceeding the natural turnover rate of the studied proteins. Interaction stability is especially challenging when the interface is under mechanical tension. Careful analysis of interface stability has allowed the use of elegant non-covalent optogenetic tools in reconstituting force-bearing proteins.^28,35^ However, local changes in force magnitude, duration, and application rate can affect bond stability and lead to inconsistent or unrepresentative results. To overcome this limitation, we developed SpyCatcher003(K31HCK) for visible-light photoactivation and demonstrated its application in the covalent reconstitution of split talin. Optical control of talin reconstitution allowed us to probe the time scale of initial adhesion complex formation, revealing biphasic extension of lamellipodia upon engagement of the adhesion clutch. We also demonstrated the use of SpyCatcher003(K31HCK) coupling over a longer time course, establishing a hierarchy of adhesion protein recruitment after engaging the adhesion clutch. The recruitment rates of adhesion proteins followed the layer structure of the adhesion complex (Figure 3D),^31,32^ suggesting that talin governs not only the nanoscale organization of the adhesion but also the timing of protein recruitment.

Light control of covalent reactivity can also be achieved with bispecific molecules regulating the bridging of SNAP-tag (19 kDa) with HaloTag (33 kDa).^36,37^ However, the larger size of this protein pair may reduce the range of accessible sites. Split intein reaction may also be regulated by photocaged tyrosine, but the reconstitution over 4 h may limit applicability for cellular processes.^38^ While this work was in progress, a related approach was reported with photocaging of the slower first-generation SpyCatcher using *o*-nitrobenzyloxycarbonyl-caged lysine.^39^ This approach used 365 nm wide-field uncaging with a 20 min uncaging time. Hence, the 405 nm-responsive amino acid used here should have lower phototoxicity for cell biology studies.^17,40−42^ Also, 405 nm lasers are common on confocal microscopes, allowing uncaging at a spatiotemporal resolution not easily achieved by using 365 nm wide-field light sources and photomasks.

Beyond adhesion, SpyCatcher003(K31HCK) may become a broadly applicable tool for the photocontrol of biomolecules. A robust cellular response was initiated in seconds here, opening possibilities for spatiotemporal control of highly dynamic intracellular and extracellular processes.

## Supporting Information Available

Western blots for the validation of SpyCatcher003(K31HCK) photoactivation (Figure S1); Western blot to validate stability of SpyCatcher003(K31HCK) in ambient light (Figure S2); phototoxicity assay for 405 nm exposure on fibroblast cells (Figure S3); cell morphology analysis for cells expressing mutated SpyTag variants (Figure S4); representative images of vinculin and VASP recruitment and raw data for adhesion protein recruitment analysis (Figure S5); methods section (PDF)
Time-lapse image series of cell spreading upon 405 nm photoactivation of talin reconstitution (Movie S1) (AVI)

## Supplementary Material

ja3c07827_si_001.pdf

ja3c07827_si_002.avi
